# Supplementary figures and images for: Genome annotation with long RNA reads reveals new patterns of gene expression and improves single-cell analyses in an ant brain
Source: BMC Biol. 2021 Nov 27;19:254. doi: 10.1186/s12915-021-01188-w (PMC8626913; doi:10.1186/s12915-021-01188-w)

**A**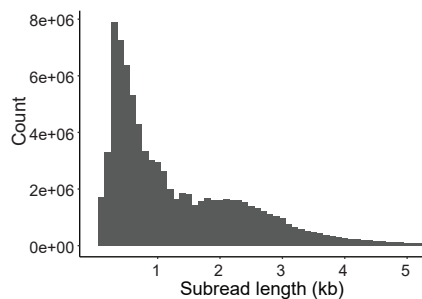**B**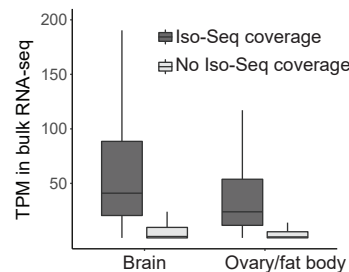**C**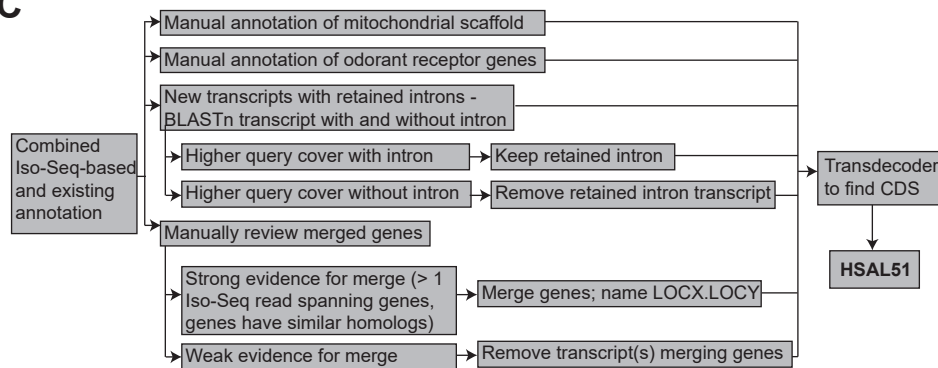**D**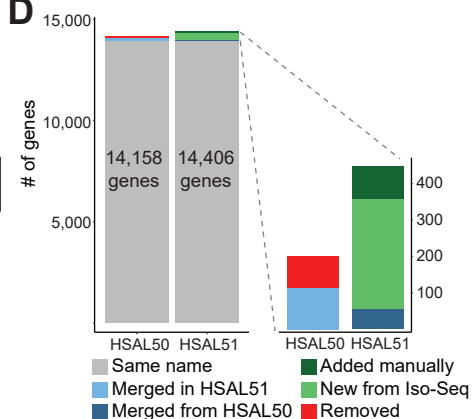**E**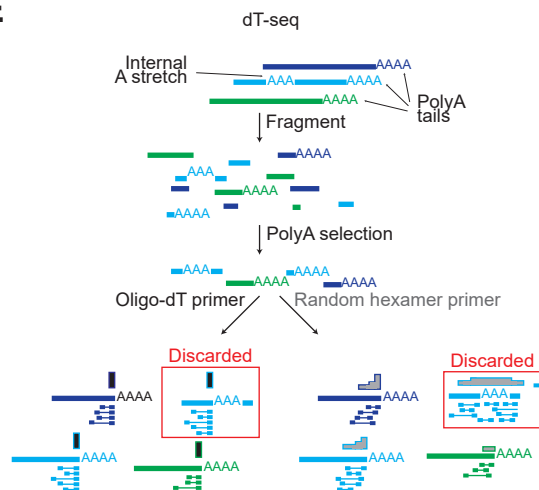**F**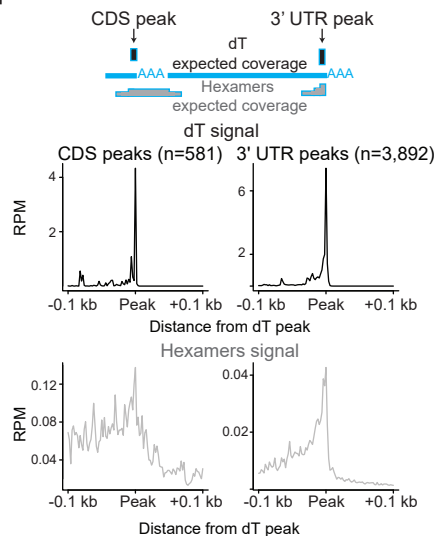

Supplement: Supplementary file 1 — Additional file 1: Figure S1. Statistics and methods used to create and evaluate the new Harpegnathos annotation. (A) Length distribution of all raw Iso-Seq subreads. (B) Transcripts per million (TPM) from short-read RNA-seq of genes with and without Iso-Seq coverage in brain and fat body/ovary. (C) Pipeline for manual annotation following combination of Iso-Seq-based and RNA-seq-based annotations. (D) Relationship between gene models in HSAL50 and HSAL51. (E) Schematic of the dT-seq approach. RNA was chemically fragmented. PolyA+ molecules were purified and split into two reverse transcription reactions, one primed with an anchored oligo-dT primer and one with random hexamers. The resulting cDNA was assembled into libraries and sequenced. The scheme at the bottom shows that the expected read distribution in dT- and hexamer-primed reactions differs for true polyA tails and internal A-stretches. This information was used to discard peaks that did not correspond to bona fide TTSs (red square). (F) Expected (top) and observed (bottom) signal at dT peaks found in the CDS (let) or 3′ UTR (right) from oligo-dT primed libraries (”dT”, top) and random hexamer primed libraries (”hexamers”, bottom). [file 12915_2021_1188_MOESM1_ESM.pdf]

**A**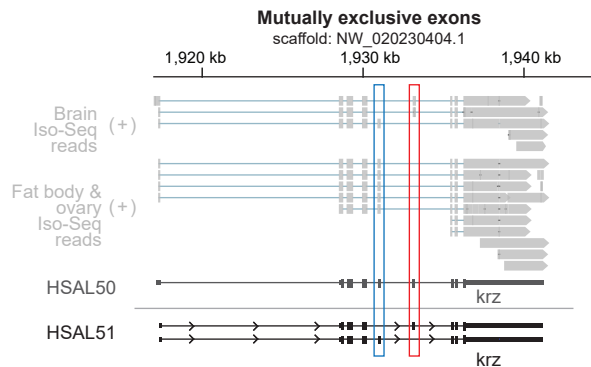**B**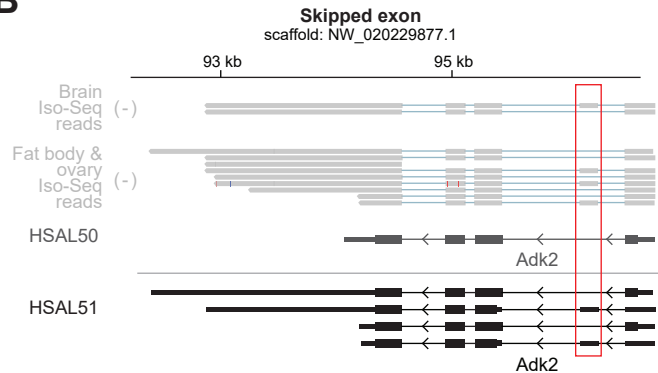**C**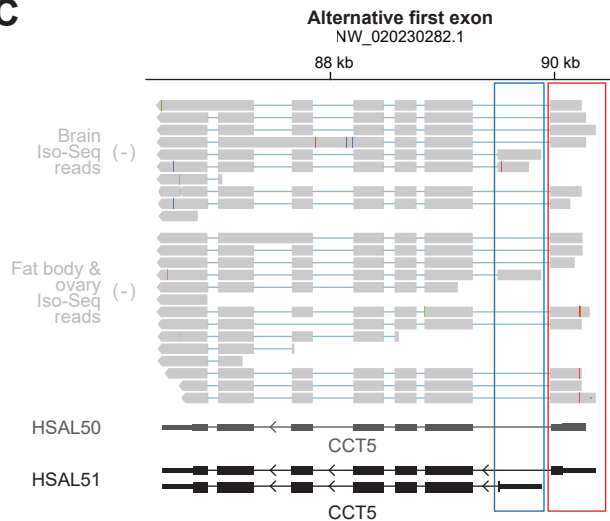**D**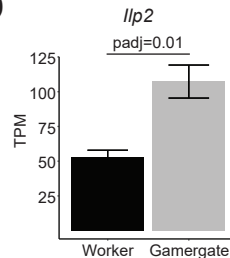**E**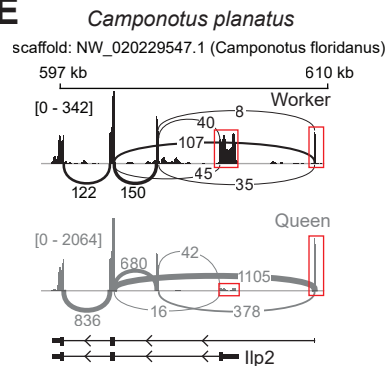**F**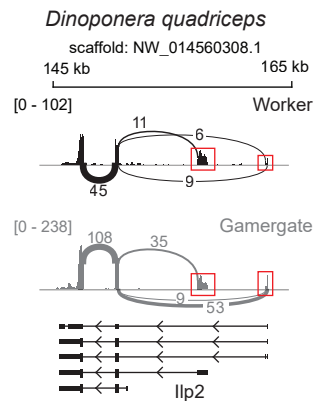

Supplement: Supplementary file 3 — Additional file 3: Figure S2. More comparisons of alternative splicing in HSAL50 and HSAL51. (A–C) Examples of a transcript with newly identified alternative splicing patterns of (A) mutually exclusive exons, (B) a skipped exon, and (C) an alternative first exon. Boxes indicate regions of the gene that is alternatively spliced. A subset of HSAL51 isoforms is shown. (D) TPM of Ilp2 (LOC105188195) in worker (n = 11) and gamergate (n = 12) brains. Padj is from DESeq2 differential expression analysis. (E) Sashimi plot for the Ilp2 gene (LOC105257206) in Camponotus planatus (RNA-seq from [32]; using Camponotus floridanus genome and annotation) for worker (n = 5) and queen (n = 5) brains. Splice junction line widths are scaled to the number of reads spanning the splice junction and the total number of reads mapped to Ilp2 for each caste. Red boxes indicate positions of first exon for each isoform. (F) Sashimi plot for the Ilp2 gene (LOC106750697) in Dinoponera quadriceps (RNA-seq from [36]) for worker (n = 6) and gamergate (n = 6) brains. Splice junction line widths are scaled to the number of reads spanning the splice junction and the total number of reads mapped to Ilp2 for each caste. Red boxes indicate positions of first exon for the two major isoforms. [file 12915_2021_1188_MOESM3_ESM.pdf]

**A**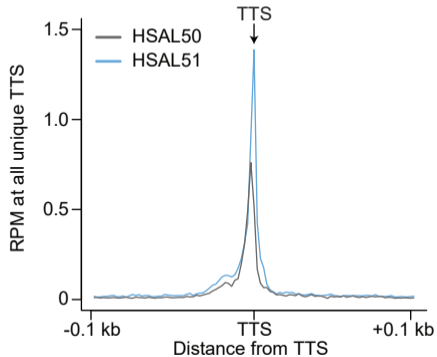**B**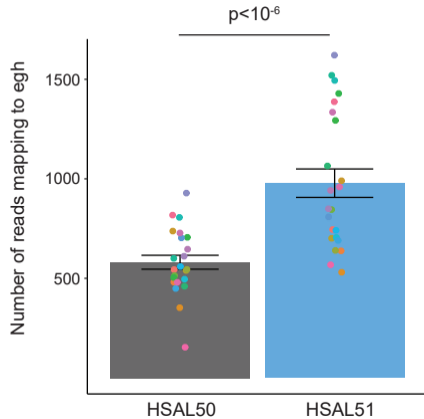

Supplement: Supplementary file 4 — Additional file 4: Figure S3. Transcript extensions and RNA-seq analysis. (A) dT-seq coverage (see Additional File 1: Fig. S1E and methods) coverage at all unique TTSs in HSAL50 (gray) and HSAL51 (blue). (B) Reads mapping to egh in HSAL50 and HSAL51. P-value is from a paired Student’s t-test. [file 12915_2021_1188_MOESM4_ESM.pdf]

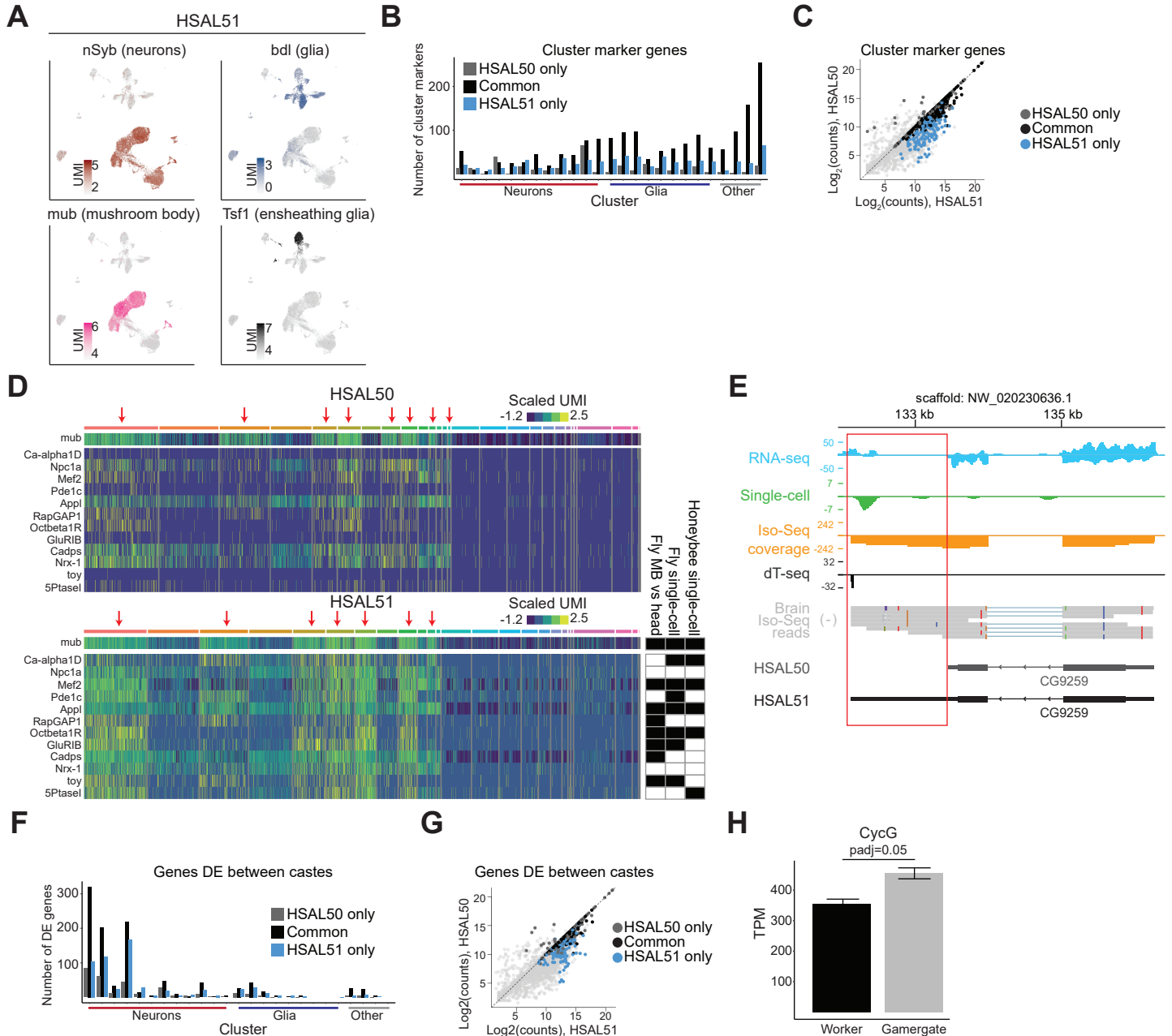

Supplement: Supplementary file 5 — Additional file 5: Figure S4. Additional single-cell analyses using HSAL50 and HSAL51 annotations. (A) Heatmaps of markers for neurons (nSyb), glia (bdl), mushroom body neurons (mub), and ensheathing glia (Tsf1) in HSAL51 single-cell clustering. (B) Number of marker genes (padj < 0.05, LFC > 1) for each cluster. Marker genes common to HSAL50 and HSAL51 analyses are shown in black, while markers unique to HSAL50 are in gray and markers unique to HSAL51 are in blue. (C) Scatter plot for UMI counts in HSAL50 vs. HSAL51 with marker genes highlighted according to (B). (D) Heatmap of newly identified mushroom body markers in HSAL51 (padj < 0.05 and logFC > 1.5). Arrows denote mushroom body clusters, as determined by mub expression (top row of heatmap). Classification of each new marker in other data sets (fly MB vs head, [43]; fly single-cell, [44]; honeybee single-cell, [45]) is indicated in heatmap to right, with black boxes indicating marker was identified as mushroom body-enriched (see methods). (E) Genome browser view showing CG9259 with RNA-seq, Iso-Seq, and single-cell coverage along with dT-seq and raw Iso-Seq reads. Scales represents counts per million. A subset of HSAL51 isoforms is shown. (F) Number of genes differentially expressed (DE) within each cluster (padj < 0.01). Differentially expressed genes common to HSAL50 and HSAL51 analyses are shown in black, while genes unique to HSAL50 are in gray and genes unique to HSAL51 are in blue. (G) Scatter plot for UMI counts in HSAL50 vs. HSAL51 with differentially expressed genes highlighted according to (F). (H) TPM of CycG in worker (n = 11) and gamergate (n = 12) brains. Padj is from DESeq2. [file 12915_2021_1188_MOESM5_ESM.pdf]

**A**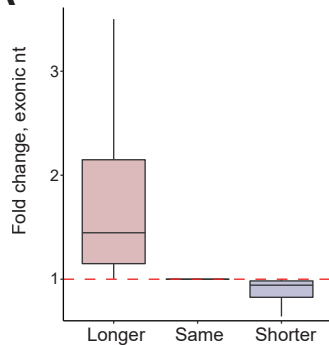**B**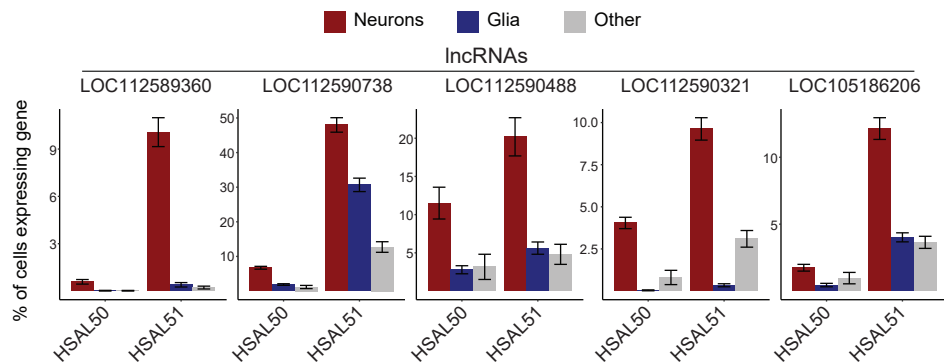**C**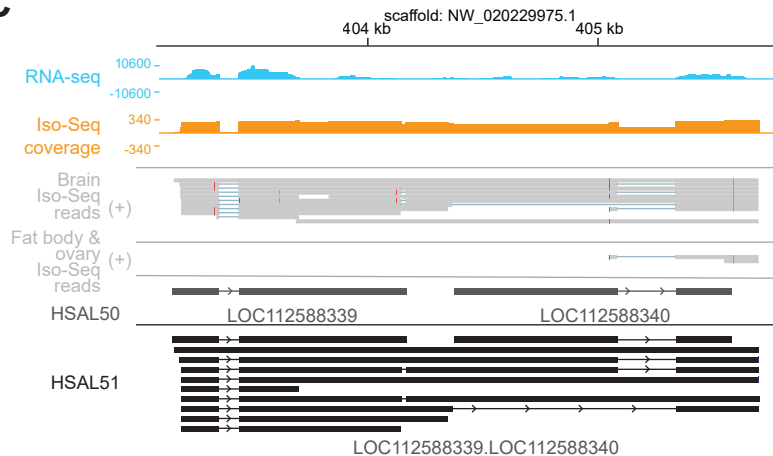**D**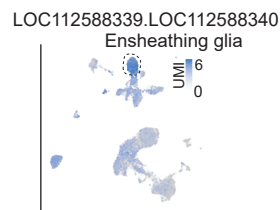**E**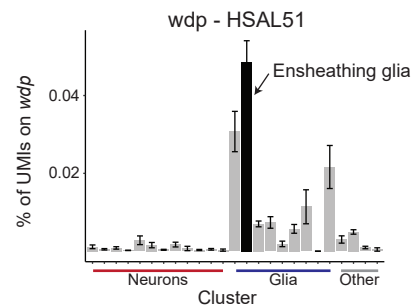

Supplement: Supplementary file 6 — Additional file 6: Figure S5. Additional single-cell analyses of lncRNA expression . (A) Fold-change in nucleotides covered by exons of lncRNAs for each category in Fig. 5A. (B) Examples of neuronal lncRNAs detected in HSAL51 showing % of neurons, glia, and other cells expressing the indicated genes in HSAL50 and HSAL51. (C) Genome browser view showing RNA-seq signal, Iso-Seq signal, and raw Iso-Seq reads of a locus containing two ensheathing glia marking lncRNAs which were merged into one gene model in HSAL51. Scales for RNA-seq and Iso-Seq represent counts per million. (D) Heatmap showing normalized UMI counts of the new merged lncRNA LOC112588339.LOC112588340 in HSAL51. (E) % of UMIs mapping to wdp in HSAL51. [file 12915_2021_1188_MOESM6_ESM.pdf]
